# Strain echocardiography identifies impaired longitudinal systolic function in patients with septic shock and preserved ejection fraction

**DOI:** 10.1186/s12947-015-0025-4

**Published:** 2015-07-02

**Authors:** Keti Dalla, Caroline Hallman, Odd Bech-Hanssen, Michael Haney, Sven-Erik Ricksten

**Affiliations:** Department of Anaesthesiology and Intensive Care Medicine, Sahlgrenska Academy, University of Gothenburg, Sahlgrenska University Hospital, Gothenburg, Sweden; Department of Clinical Physiology, Sahlgrenska Academy, University of Gothenburg, Sahlgrenska University Hospital, Gothenburg, Sweden; Department of Anaesthesiology and Intensive Care Medicine, Umeå University, Umeå, Sweden

**Keywords:** Sepsis, Ventricular function, Strain echocardiography, Myocardial dysfunction, Adults

## Abstract

**Background:**

Myocardial dysfunction is recognized in sepsis. We hypothesized that mechanical left (LV) and right (RV) ventricular function analysed using 2-dimensional speckle-tracking echocardiography in a cohort of early severe sepsis or septic shock patients, would be different to that of a group of critically ill, non-septic patients.

**Methods:**

Critically ill adult patients with early, severe sepsis/septic shock (*n* = 48) and major trauma patients with no sepsis (*n* = 24) were included retrospectively, as well as healthy controls (*n* = 16). Standard echocardiographic examinations, including right (RV) left (LV) volumes and mitral, aortic and pulmonary vein Doppler flow profiles, were retrospectively identified and the studies were then reanalysed for assessment of myocardial strain using speckle-tracking echocardiography. Endocardial tracing of the LV was performed in apical four-chamber (4-Ch), two-chamber (2-Ch), apical long-axis (3-Ch) and apical views of RV determining the longitudinal LV and RV free wall strain in each subject.

**Results:**

In septic patients, heart rate was significantly higher (*p* = 0.009) and systolic (*p* < 0.001) and mean arterial pressures (*p* < 0.001), as well as systemic vascular resistance (*p* < 0.001) were significantly lower when compared to the non-septic trauma group. Ninety-three per cent of the septic patients and 50 % of the trauma patients were treated with norepinephrine (*p* < 0.001). LV ejection fraction (LVEF) was lower in the septic patients (*p* = 0.019). In septic patients with preserved LVEF (>50 %, *n* = 34), seventeen patients (50 %) had a depressed LV global longitudinal function, defined as a LV global strain > −15 %, compared to two patients (8.7 %) in the non-septic group (*p* = 0.0014). In septic patients with preserved LVEF, LV global and RV free wall strain were 14 % (*p* = 0.014) and 17 % lower (*p* = 0.008), respectively, compared to the non-septic group with preserved LVEF. There were no significant differences between groups with respect to LV end-diastolic or end-systolic volumes, stroke volume, or cardiac output. There were no signs of diastolic dysfunction from the mitral or pulmonary vein Doppler profiles in the septic patients.

**Conclusions:**

LV and RV systolic function is impaired in critically ill patients with early septic shock and preserved LVEF, as detected by Speckle-tracking 2D echocardiography. Strain imaging may be useful in the early detection of myocardial dysfunction in sepsis.

**Electronic supplementary material:**

The online version of this article (doi:10.1186/s12947-015-0025-4) contains supplementary material, which is available to authorized users.

## Background

Patients with severe sepsis or septic shock often have significant myocardial dysfunction. A common and often reversible pattern of ventricular dilation, depressed systolic function, though with maintained or increased cardiac output has been described [1–4]. On the other hand, there have been no readily available non-invasive or minimally invasive tools, which can identify ventricular dysfunction early in the course of sepsis, before ejection fraction and cardiac output change.

In more recent years, 2-dimensional (2D) echocardiography has been used to study myocardial dysfunction in severe sepsis, demonstrating impaired LV function in septic shock and a high incidence of global LV hypokinesia with LV dilatation as well as, in some patients, isolated impairment of LV relaxation [5–8]. Myocardial function in sepsis has been evaluated with tissue Doppler imaging (TDI) measuring systolic and diastolic mitral annular velocities [9–13]. Early diastolic mitral annular velocity can be reduced in septic patients [9–11], as a TDI sign of diastolic dysfunction. A delayed early myocardial relaxation has been shown to be a strong predictor of mortality [9], while others have shown that a high peak systolic annular velocity (>9 cm/s) is an independent predictor of 90-days mortality in septic shock patients [12, 13].

Myocardial deformation during systole or diastole may provide a more specific indication of global myocardial dysfunction [14]. In a recent study, using a large animal model of severe sepsis, strain echocardiography revealed myocardial systolic dysfunction, as assessed by a fall in longitudinal myocardial strain, before significant changes in cardiac output or LV ejection fraction were seen [15].

The aim of the present study was to evaluate myocardial function in patients with early severe sepsis or septic shock using 2D speckle tracking strain echocardiography. Another cohort of critically ill patients (major trauma) with no sepsis, served as a control group, to see if there are early strain indicators of LV dysfunction related to sepsis. We tested the hypothesis that 2D speckle tracking imaging may detect LV systolic dysfunction not diagnosed by conventional echocardiography in early clinical severe sepsis and that these findings were not present in critically ill non-septic patients.

## Methods

### Study population

This study was approved by the Gothenburg Regional Human Ethics Committee (Dnr: 580–14), which waived the need for informed consent, because of the retrospective analysis of anonymised patients. From the first of January 2011 to the 10th of December 2014, conventional echocardiograms were retrospectively reviewed in adult (>18 years) septic and critically ill major trauma patients with no sepsis. The septic patients met American College of Chest Physicians/Society of Critical Care Medicine criteria for severe sepsis or septic shock [16]. The indication for echocardiography was hemodynamic instability and echocardiograms were performed within 48 h after arrival to the intensive care unit (ICU) in all patients. Exclusion criteria included previous history of ischemic cardiac disease, presence of congestive heart failure, moderate to severe valvular heart disease, arrhythmias and poor echocardiographic image quality. Additionally, as a reference group, normal echocardiograms were obtained from the institutional echocardiographic database. These healthy controls (*n* = 16) were matched by gender and age to the septic patients.

Blood pressures were obtained from arterial and central venous direct measurements. According to the institutional protocol, norepinephrine was used to treat hypotension (systolic arterial pressure < 90 mmHg) in volume-resuscitated patients. Milrinone or epinephrine was used for inotropic support at the discretion of the attending physician. Mechanical ventilation was performed with a tidal volume of 6–7 ml/kg body weight minute ventilation to obtain normocarbia and positive end-expiratory pressure (PEEP) level set by the attending physician (at least 5 cm H_2_O). The inspired oxygen fraction (FiO_2_) was adjusted to obtain an arterial oxygen saturation of > 90 %. Peak inspiratory pressure, tidal volume, respiratory rate, arterial oxygen saturation (SaO_2_), fraction of O_2_ in inspired air (FiO_2_) and end-tidal carbon dioxide were recorded. During mechanical ventilation, the patients were sedated with propofol and fentanyl infusions.

In each patient, hemodynamic and respiratory variables were obtained from review of the patient’s intensive care chart at the time of the echocardiographic examination. The following respiratory parameters were recorded: fractional inspired oxygen, peak inspiratory pressure, positive end-expiratory pressure, and oxyhaemoglobin saturation. The following hemodynamic variables were recorded: systolic arterial and mean arterial (MAP) pressures and central venous pressures (CVP), Systemic vascular resistance (SVR) was calculated as (MAP-CVP)/cardiac output. The simplified acute physiology score was recorded in all patients on arrival to the ICU and injury severity score was recorded in the trauma patients. ICU mortality, 30-days mortality, ICU length of stay, cause of sepsis and the results of blood or local site cultures were recorded in all patients.

### Standard echocardiographic examination

Echocardiograms were obtained using one of three different ultrasound machines (Vivid E9, GE Healthcare, USA, iE33, Philips Healthcare, Netherlands and X300, Siemens, Germany). Standard measurements of LV systolic and RV systolic function in all subjects included LV volumes, ejection fraction (modified Simpson’s rule), LV and RV fractional area change (apical four chamber view), time velocity integral in the LV outflow tract (TVI-LVOT) and stroke volume (π x LVOT radius^2^ x TVI-LVOT) [17] were calculated. Mitral, aortic and pulmonary vein Doppler flow profiles were recorded for measurements of LV isovolumic relaxation time, peak early LV diastolic flow deceleration time (E-deceleration time), maximum flow velocity during LV early (E) and late (A) diastolic filling and pulmonary vein peak systolic (S) and peak diastolic (D) flow velocities. The ratios of E/A and S/D were calculated.

### Strain echocardiography

Echocardiograms were stored in a digital format (DICOM) in the hospitals PAX system. The investigations were retrieved in DICOM format with preserved frame rate and transferred to and analysed in an offline system (Syngo Velocity Imaging System, Siemens, Germany). Myocardial strain is defined as a fractional change in length between 2 time points, end-diastole (L_0_) and end-systole (L) and calculated as: (L-L_0_)/L or ∆L/L_0_. Strain is presented as percent change (%). Negative values of strain indicate myocardial contraction. A frame rate of 52 ± 3 frames/s and 50 ± 3 were obtained for the LV and RV, respectively.

The peak longitudinal systolic strain was determined for the RV using the four-chamber view and presented as the mean of the three segments of the free wall. The peak longitudinal LV strain was determined using the three apical projections and presented as the mean of the 18 segments.

A subgroup of patients with a preserved LV ejection fraction (PEF), that is an EF > 50 %) in the sepsis group (*n* = 34, sepsis-PEF) and the trauma group (*n* = 23, trauma-PEF), were analysed separately.

One investigator analysed both the conventional and strain echocardiographic examinations, blinded to the diagnoses.

### Statistical analysis

The primary endpoint in the study was the comparison of ventricular systolic strain (right and left ventricles) between the sepsis and the trauma groups (all with preserved EF). Including at least 20 patients in each group gave us a power of 0.89 to detect a 20 % difference in global systolic strain between groups at a α = 0.05 and a standard deviation of 3.0. Differences between septic and trauma patients were compared using independent Student’s t-test. Categorical baseline data were compared using Fisher’s exact test. A linear regression analysis was applied to quantify the strength of the relationship between global LV systolic strain and LV ejection fraction for septic and trauma patients. A multivariate analysis was performed to identify variables that were independent predictors of global LV systolic strain. The following independent variables were included in the model: end-diastolic volume, (preload), systolic arterial pressure (afterload) and heart rate or the diagnosis (sepsis or trauma). The coefficients of variation for intra-observer agreement for paired observations of LV systolic strain in apical four-chamber (4-Ch), two-chamber (3-Ch), apical long-axis (2-Ch) views were calculated. The data are presented as mean ± standard deviation (SD). A probability level (p-value) of less than 0.05 was considered to indicate statistical significance.

## Results

Two-hundred and eighty-nine patients were treated at the ICU for severe sepsis/septic chock during the study period. A conventional echocardiogram examination was performed because of haemodynamic instability in 135 patients. Forty-eight patients fulfilled inclusion but not exclusion criteria and were evaluated together with 24 major trauma patients. Baseline patient characteristics are shown in Table 1. There were no significant differences between these groups with respect to gender, body weight, need for mechanical ventilation or ICU length of stay. Sepsis patients were older, evaluated slightly later after their ICU admission and had a higher 30-day mortality. The SAPS II score was significantly higher in the septic patients. Pneumonia was the most common cause of sepsis, followed by abdominal sepsis, necrotising fasciitis, and urosepsis (all diagnoses shown in Additional file 1: Table S1).

**Table 1:** Patient characteristics

	Sepsis	Trauma	p-value	Sepsis	Trauma	p-value
	All (*n* = 48)	(*n* = 24)		EF ≥ 50 % (*n* = 34)	EF ≥ 50 % (*n* = 23)	
Age (years)	54 ± 14	40 ± 17	0.001	53 ± 14	41 ± 16	0.006
Male sex, n (%)	30 (62)	19 (79)	0.153	22 (65)	18 (78)	0.272
Weight, kg	77.1 ± 15.6	80.3 ± 13.9	0.386	78.7 ± 14.8	79.9 ± 14.1	0.763
Study day from ICU arrival	1.4 ± 0.7	1.8 ± 0.9	0.040	1.3 ± 0.6	1.9 ± 1.0	0.010
Mechanical ventilation, n (%)	38 (79)	15 (63)	0.130	26 (76)	15 (65)	0.354
SAPS II on ICU arrival	68.3 ± 14	44.6 ± 13	<0.001	67 ± 15	44 ± 14	<0.001
Injury Severity Score		32 ± 19			32 ± 19	
ICU length of stay	10.7 ± 10	6.6 ± 7.8	0.081	10.3 ± 11	6.8 ± 8	0.191
ICU mortality (%)	14	8.3	0.450	5 (15)	1 (4)	0.211
30-day mortality (%)	27	8.3	0.065	8 (24)	1 (4)	0.051
Cause of sepsis, n (%)						
Pulmonary infection, n (%)	27 (56)			19 (56)		
Necrotising fasciitis, n (%)	4 (8)			3 (9)		
Urosepsis, n (%)	3 (6)			3 (9)		
Abdominal sepsis, n (%)	11 (23)			7 (21)		
Other, n (%)	3 (6)			2 (6)		

Data are presented as means ± SD, SAPS II; Simplified Acute Physiology Score, ICU; Intensive Care Unit

### Hemodynamic data (Table 2)

**Table 2:** Hemodynamic characteristics

Variables	Sepsis	Trauma	p-value	Sepsis	Trauma	p-value
	All (*n* = 48)	All (*n* = 24)		EF ≥ 50 % (*n* = 34)	EF ≥ 50 % (*n* = 23)	
Heart Rate (bpm)	103 ± 20	89 ± 23	0.009	102 ± 19	89 ± 23	0.023
Systolic arterial pressure (mmHg)	106 ± 14	123 ± 17	<0.001	108 ± 14	123 ± 17	0.001
Mean arterial pressure (mmHg)	71 ± 9	79 ± 10	<0.001	72 ± 9	79 ± 10	0.020
Central venous pressure (mmHg)	12 ± 5	9 ± 3	0.003	11 ± 5	9 ± 3	0.109
SVR (dynes x s/cm^−5^)	905 ± 294	1129 ± 188	<0.001	958 ± 279	1123 ± 189	0.017
Right Ventricular Systolic Pressure	40.8 ± 19.1	35.6 ± 7.9	0.169	42 ± 10	35 ± 8	0.138
Vasoactive drugs n (%)						
Norepinephrine, n (%)	45 (93)	12 (50)	<0.001	32 (94)	11 (48)	**<**0.001
Milrinone, n (%)	3 (6)	0	0.476	1 (3)	0	0.407
Adrenaline, n (%)	8 (16)	0	0.034	5 (15)	0	0.054

Data are presented as means ± SD, SVR; systemic vascular resistance

Heart rate, was higher whereas systolic and mean arterial pressures, as well as SVR were significantly lower when compared to the non-septic trauma group. The CVP was significantly higher in the sepsis group (all) compared to the trauma group (all), while CVP did not differ significantly comparing sepsis-PEF to trauma-PEF groups. There was no difference in right ventricular systolic pressure between groups. The use of the vasopressor therapy (norepinephrine or epinephrine) was more frequent in the sepsis group.

### Respiratory variables (Table 3)

**Table 3:** Respiratory variables

	Sepsis	Trauma	p-value	Sepsis	Trauma	p-value
	All (*n* = 38)	All (*n* = 15)		EF ≥ 50 % (*n* = 26)	EF ≥ 50 % (*n* = 15)	
PEEP (cm H_2_O)	10.2 ± 3.0	8.3 ± 2.5	0.037	10.2 ± 3.2	8.4 ± 2.7	0.065
Peak inspiratory pressure (cm H_2_O)	25.9 ± 5.0	21.6 ± 4.7	0.007	26.6 ± 5.3	21.8 ± 4.8	0.007
Tidal volume (ml)	480 ± 97	487 ± 52	0.787	482 ± 101	491 ± 51	0.887
Respiratory rate	20.6 ± 5.5	17.5 ± 2.9	0.005	20.8 ± 5.6	17.6 ± 3.0	0.029
Arterial oxygen saturation (%)	95.9 ± 2.8	98.2 ± 1.0	<0.001	96 ± 2.4	98 ± 1.0	0.001
Fraction of inspired oxygen	0.51 ± 0.2	0.34 ± 0.13	<0.001	0.52 ± 0.2	0.34 ± 0.13	<0.001
End-tidal carbon dioxide (%)	4.8 ± 1.4	4.9 ± 0.5	0.712	4.7 ± 1.2	5.0 ± 0.4	0.392

Data are presented as means ± SD, PEEP; positive end-expiratory pressure

Peak inspiratory and positive end-expiratory pressures and respiratory rate were higher in the septic group, while there was no difference in tidal volumes between groups. A higher mean FiO_2_ and a lower SaO_2_ were seen in the septic group compared to the trauma group. End-tidal carbon dioxide did not differ between groups.

### Conventional echocardiography (Table 4)

**Table 4:** Conventional echocardiographic variables

	Healthy controls	Sepsis	Trauma	p-value	Sepsis	Trauma	p-value
	(*n* = 16)	All (*n* = 48)	All (*n* = 24)	sepsis vs trauma	EF ≥ 50 % (*n* = 34)	EF ≥ 50 % (*n* = 23)	sepsis vs trauma
LV end-diastolic volume (ml)	107 ± 19	92 ± 29	96 ± 20	0.500	89 ± 20	97 ± 21	0.174
LV end-systolic volume (ml)	40 ± 8	41 ± 18	38 ± 11	0.501	36 ± 12	38 ± 11	0.491
LV ejection fraction (%)	63 ± 4	54 ± 12	60 ± 7	0.019	60 ± 8	60 ± 6	0.973
LV fractional shortening (%)	59 ± 4	49 ± 12	55 ± 9	0.066	49 ± 12	55 ± 9	0.832
RV fractional area change (%)	47 ± 6	40 ± 16	45 ± 26	0.067	54 ± 10	55 ± 9	0.156
Stroke volume (ml)	84 ± 20	68 ± 22	70 ± 14	0.711	74 ± 20	71 ± 13	0.457
TVI-LVOT (cm)	22 ± 3	17.5 ± 5	18.6 ± 4	0.356	19.2 ± 4.4	18.9 ± 4.0	0.802
Cardiac output (l/min)	5.6 ± 1.5	6.7 ± 2.3	6.0 ± 1.9	0.273	7.2 ± 2.1	6.2 ± 1.8	0.070
LV IVRT (ms)	82 ± 24	65 ± 23	68 ± 20	0.603	63 ± 23	68 ± 20	0.437
E-deceleration time (ms)	193 ± 38	175 ± 59	172 ± 55	0.845	177 ± 66	172 ± 55	0.746
E (cm/sek)	72 ± 16	77 ± 30	69 ± 15	0.262	80 ± 34	69 ± 15	0.969
A (cm/sek)	54 ± 15	60 ± 28	54 ± 15	0.357	65 ± 28	54 ± 15	0.183
E/A	1.3 ± 0.4	1.5 ± 0.8	1.3 ± 0.4	0.342	1.3 ± 0.3	1.3 ± 0.4	0.122
S/D	1.1 ± 0.2	0.9 ± 0.3	0.9 ± 0.3	0.939	1.0 ± 0.3	0.9 ± 0.3	0.623

Data are presented as means ± SD. *LV* left ventricular; *RV* right ventricular; *TVI-LVOT* time velocity integral of the LV outflow tract; *E-deceleration time* peak early LV diastolic flow deceleration time; *E* maximum flow velocity during early LV diastolic filling; *A* maximum flow velocity during late diastolic LV filling, S/D; ratio between pulmonary vein peak systolic (S) and peak diastolic (D) flow velocities

**Fig. 1:**
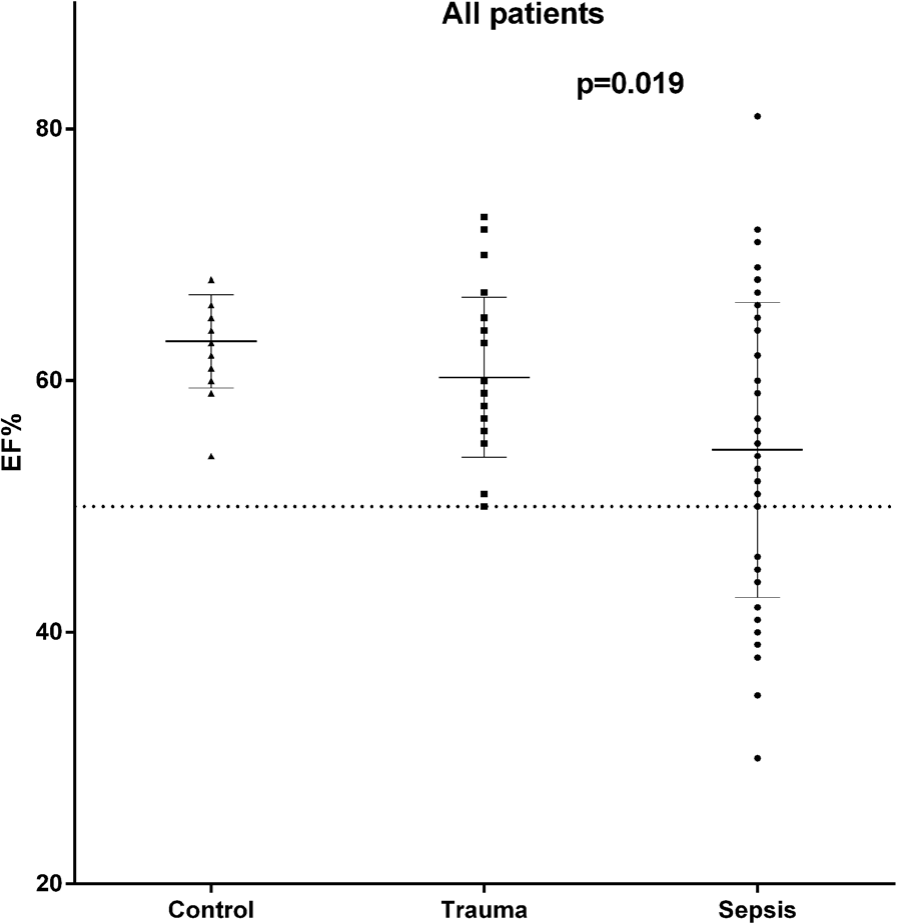
Shows the individual data on left ventricular ejection fraction (EF) as assessed by conventional echocardiography in healthy controls and in patients with trauma or sepsis. The dashed line denotes the cut-off value for normal/abnormal left ventricular ejection fraction (50 %)

LV ejection fraction was slightly but significantly (*p* = 0.019) (Fig. 1) lower and there was also a trend for a lower LV fractional shortening in the septic (all) compared to the trauma group (all) (*p* = 0.066). There were no significant differences between the sepsis (all) and the trauma (all) group with respect to stroke volume, TVI-LVOT or cardiac output. The diastolic function variables did not differ between the septic and then trauma patients. There were no differences in any of the measured conventional echocardiographic variables comparing the sepsis-PEF with the trauma-PEF group, although there was a trend for a higher cardiac output in the sepsis-PEF group (*p* = 0.070).

There was a trend for a lower RV fractional area change in the sepsis group (all) compared to the trauma group (all) (*p* = 0.067). There were no differences in any of the measured conventional echocardiographic variables comparing the sepsis-PEF with the trauma-PEF group

### Strain echocardiography (Table 5)

**Table 5:** Strain echocardiographic variables

	Healthy controls	Sepsis	Trauma	p-value sepsis vs trauma	Sepsis	Trauma	p-value sepsis vs trauma
	(*n* = 16)	All (*n* = 48)	All (*n* = 24)		EF ≥ 50 % (*n* = 34)	EF ≥ 50 % (*n* = 23)	
LV strain (4-Ch) (%)	−18.2 ± 2.3	−14.5 ± 4.4	−16.4 ± 2.4	0.028	−15.4 ± 4.6	−16.5 ± 2.4	0.278
LV strain (2-Ch) (%)	−18.9 ± 2.3	−13.8 ± 4.5	−17.7 ± 4.2	0.001	−14.9 ± 4.5	−17.7 ± 4.2	0.027
LV strain (3-Ch) (%)	−19.3 ± 1.9	−14.2 ± 4.3	−17.9 ± 3.9	0.001	−14.8 ± 4.6	−17.8 ± 3.9	0.018
LV global systolic strain (%)	−18.8 ± 1.6	−14.1 ± 3.8	−17.3 ± 2.8	<0.001	−14.9 ± 4.0	−17.3 ± 2.9	0.014
RV lateral free wall systolic strain (%)	−28.8 ± 2.8	−19.5 ± 5.4	−24.7 ± 5.0	<0.001	−20.8 ± 5.5	−25.0 ± 5.0	0.008

Data are presented as means ± SD. *LV* left ventricular; *Ch* chamber; *RV* right ventricular

The coefficient of variation for intra-observer agreement for paired observations of LV systolic strain in apical four-chamber (4-Ch), two-chamber (3-Ch), apical long-axis (2-Ch) and RV free wall views, were 9.0 %, 11.0 %, 14 % and 9.6 %, respectively. The corresponding data for inter-observer agreement were: 8.6 %, 9.5 %, 8.3 % and 8.8 %, respectively.

**Fig. 2:**
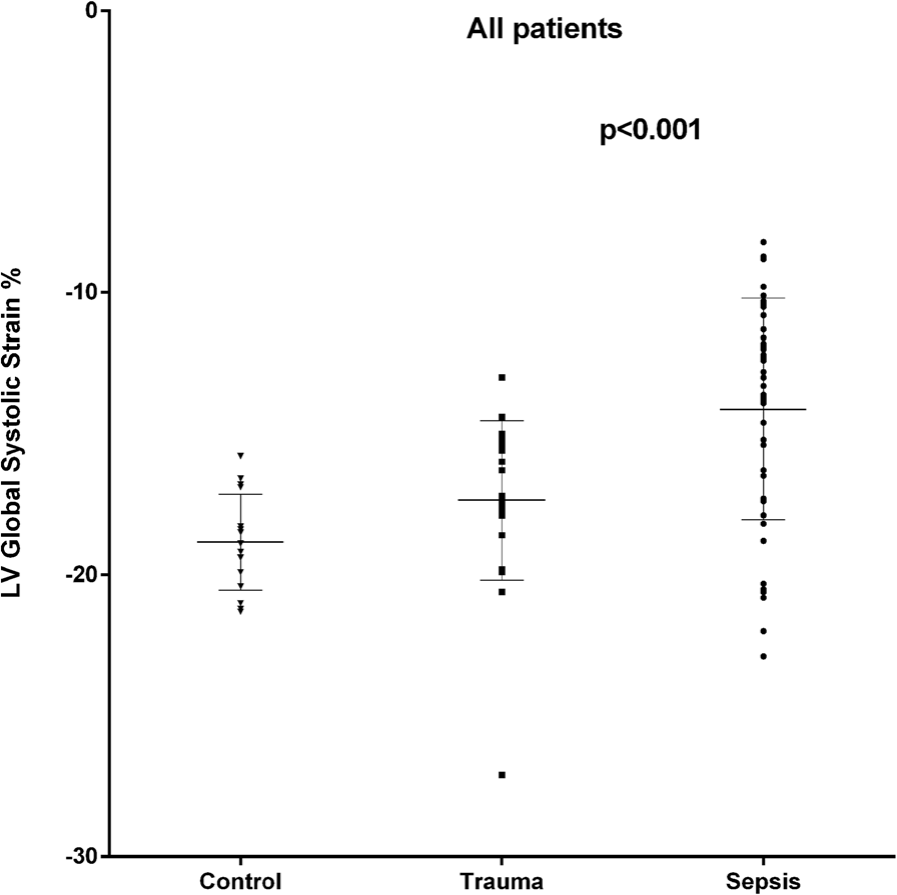
Shows the individual data on global longitudinal left ventricular strain as assessed by speckle tracking echocardiography in healthy controls and in all patients with trauma (*n* = 24) or sepsis (*n* = 48)

**Fig. 3:**
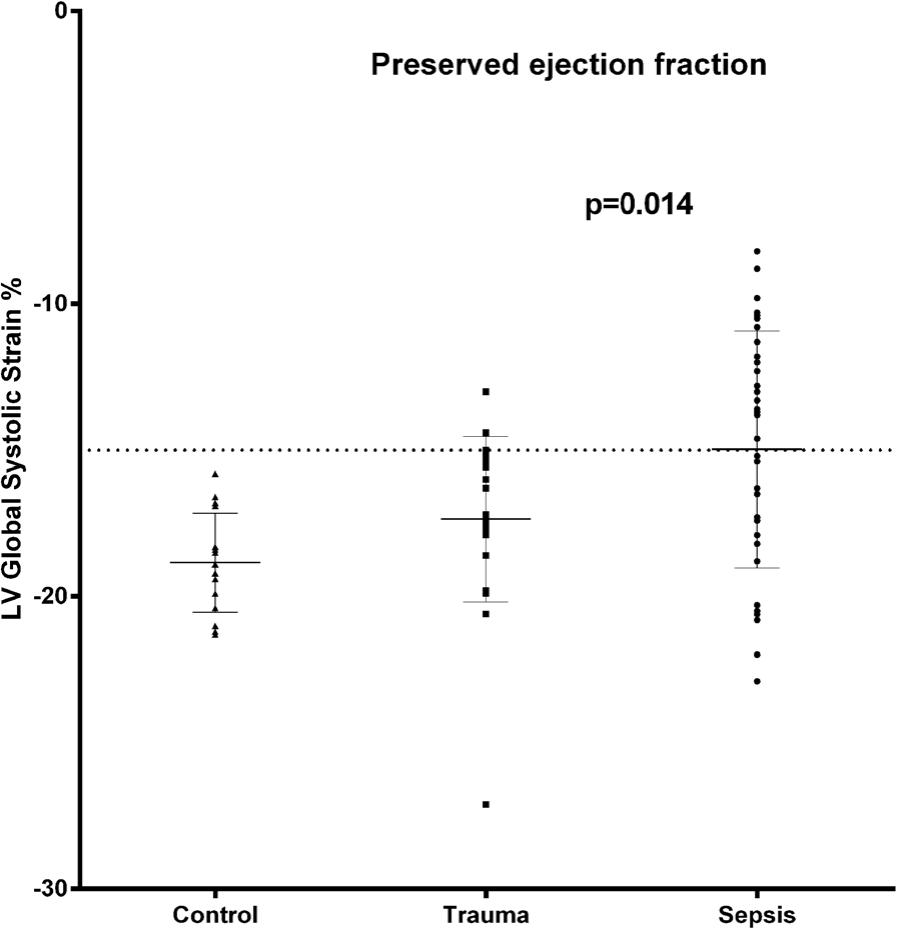
Shows the individual data on global longitudinal left ventricular strain as assessed by speckle tracking echocardiography in healthy controls and in patients with trauma (*n* = 23) or sepsis (*n* = 34) and preserved left ventricular ejection fraction. The dashed line denotes the cut-off value for normal/abnormal left ventricular longitudinal strain as used in the present study (−15 %)

**Fig 4:**
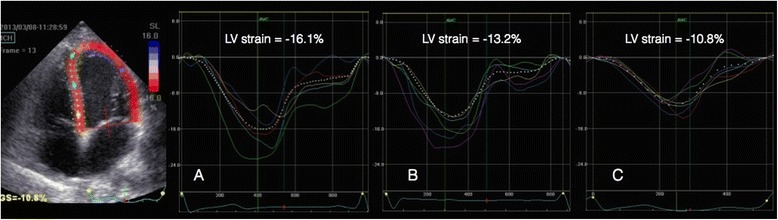
Shows 4-chamber recordings of longitudinal left ventricular strain from a healthy individual (**a**), one trauma patient (**b**) and a septic patient (**c**). Both patients had preserved left ventricular function

LV strain was significantly lower in all three apical views and the LV global longitudinal strain was 18 % lower in the septic (all) compared to the trauma group (all) (Fig. 2). The correlation coefficients between LV global longitudinal strain and LV ejection fraction were 0.43 (*p* = 0.003) and 0.39 (*p* = 0.061) for the sepsis (all) and the trauma group (all), respectively. In the sepsis-PEF group, the LV strain was significantly lower in the 2-Ch and 3-Ch views and the LV global strain was 14 % lower compared to the trauma-PEF group (Figs. 3 and 4). Two of the trauma-PEF (8.7 %) and seventeen of the sepsis-PEF patients (50 %) had a depressed LV function, defined as a LV global strain > −15 % (*p* = 0.0014).

**Fig. 5:**
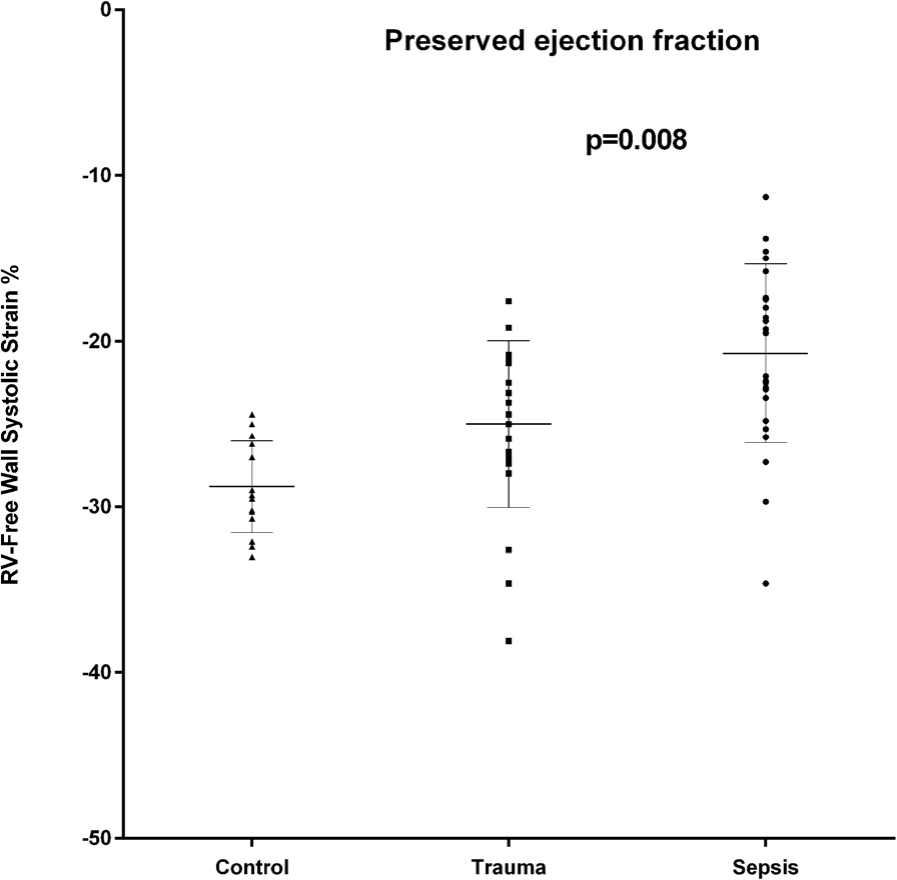
Shows the individual data on right ventricular free wall strain as assessed by speckle tracking echocardiography in healthy controls and in patients with trauma (*n* = 23) or sepsis (*n* = 34) and preserved left ventricular ejection fraction

**Fig. 6:**
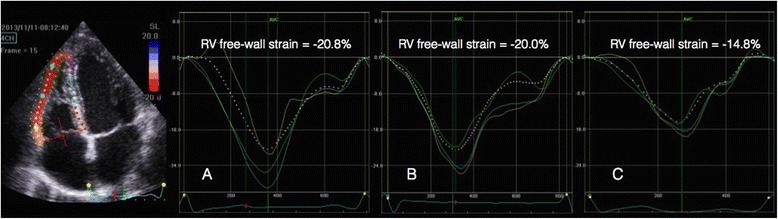
Shows 4-chamber recordings of RV free wall longitudinal strain from a healthy individual (**a**), one trauma patient (**b**) and a septic patient (**c**). Both patients had preserved left ventricular function

RV strain was 21 % lower in the septic (all) compared to the trauma group (all) and 17 % lower in the sepsis-PEF compared to the trauma-PEF (Figs. 5 and 6).

### Multivariate analysis (Table 6)

**Table 6:** Multivariate analysis variables independently associated with global LV strain

	Coefficient (ß)	95 % CI for ß	t-value	p-value
Sepsis or trauma	2.8	0.64–4.96	2.59	0.012
Systolic arterial pressure	−0.013	−0.07–0.04	−0.456	0.650
End-diastolic volume	−0.007	−0.04–0.03	−0.395	0.694
Heart rate	−0.056	−0.09–(−0.02)	−2.718	0.009

This analysis revealed that heart rate (*p* = 0.009, ß = −0.056, 95 % CI: −0.09–(−0.02)), but not end-diastolic volume or systolic arterial pressure, was independently associated with global LV strain. Furthermore, the diagnosis (sepsis or trauma) could predict global LV strain (*p* = 0.012, ß = 2.8, 95 % CI: 0.64-4.96).

## Discussion

We assessed myocardial function in critically ill adult patients with early severe sepsis or septic shock, as well as major trauma, using strain echocardiography. The main findings were that LV and RV systolic performances, as detected by speckle tracking imaging (STI), were impaired to a greater extent in septic patients with preserved ejection fraction, when compared to critically ill, non-septic, trauma patients with preserved ejection fraction.

For the clinical application of speckle tracking echocardiography, the definition of normal values of LV strain is of crucial importance. However, the results from speckle tracking are known to be vendor-dependent and the lower border of normality is not defined [18]. In a recent meta-analysis, the normal range of LV global longitudinal strain, obtained by transthoracic echocardiography, was found to be −15.9 to −22.1 % [19]. In the present study we therefore used a cut-off value of −15 %, supported by both the findings in our own group of healthy controls and the meta-analysis [19], to define depressed global longitudinal LV function and found that a large proportion (50 %) of septic patients with preserved ejection fraction had depressed longitudinal LV function, compared to 8.7 % in critically ill non-septic patients. Thus, STI and ventricular strain analysis can detect impaired LV systolic function in early sepsis that may be missed by conventional echocardiography.

Our data are in line with a recent study on myocardial performance in children with septic shock using STI echocardiography [20]. They found that both longitudinal and circumferential strain was reduced in septic children, while there was no difference in LV ejection fraction or fractional shortening between septic patients or healthy controls. In a recent experimental pig model of severe E-coli sepsis, Hestenes et al. [15] demonstrated that during sepsis, LV longitudinal strain decreased in spite of a decreased afterload. Similar findings were demonstrated in the present study, in which longitudinal myocardial strain was lower in the sepsis group compared with controls and trauma patients. This lower longitudinal strain, despite lower systolic blood pressure and lower systemic vascular resistance, as well as a more frequent use of catecholamines, strongly suggests that LV systolic function was impaired in the septic patients.

In healthy subjects it was recently shown that free wall RV strain by STI is between −27 % and −30 % [21, 22]. In the present study, free RV free wall strain was −29 % in healthy controls, −25 % in the trauma group and −20 % in the septic patients. To our knowledge, this is the first study comparing septic to non-septic critically ill patients with respect to RV function using speckle-tracking echocardiography. In the experimental study by Hestenes et al. [15], the pronounced decrease in RV strain was explained not only by a direct myocardial depression but also by a sepsis-induced, 4-fold increase in pulmonary vascular resistance. In the present study, the RV systolic strain was clearly lower in the septic patients compared to the trauma patients. This lower RV strain could be caused by a higher RV afterload, as more patients in this group were mechanically ventilated with higher airway pressure and with more pronounced lung injury compared to the trauma patients. RV systolic pressure did, however, not differ between groups. The most likely explanation for the lower RV strain in the septic group is, therefore, the septic process itself that causes a septic cardiomyopathy involving both LV and RV.

Using mitral annular TDI, it has been suggested that diastolic function is impaired in septic shock [9, 11]. When active LV relaxation is impaired but the LV filling pressure is normal, the mitral Doppler flow indices E velocity, E/A ratio decreases and the ratio between pulmonary vein peak systolic and peak diastolic flow velocities typically increase. Furthermore, these changes will be reinforced by increases in heart rate, which will, per se, redistribute LV filling to late diastole, resulting in an increase in A velocity and a decrease in the E/A ratio [23, 24]. As the patients in the septic group were older and had higher heart rates, one would have expected lower E/A ratios in this group. In the present study, however, there was no difference in mitral Doppler flow profiles, which speaks against early diastolic dysfunction in the septic compared to the trauma patients.

When evaluating RV and LV function in critically ill septic patients, the choice of a control group is critical. In ICU patients, with on-going multimodal intensive care treatment including sedation, mechanical ventilation, fluid therapy, vasoactive and inotropic treatment as well as, renal replacement therapy, one must consider the possibility that these treatment modalities themselves may affect RV and LV function. It has been shown that positive pressure ventilation with PEEP causes a decrease in RV strain and cardiac chambers’ volume [25]. In the present study, we therefore chose to include as controls critically ill non-septic patients requiring intensive care treatment after major trauma. This patient group demonstrates some degree of systemic inflammatory response syndrome, which itself is recognized to cause myocardial dysfunction [26]. Major trauma is defined as an injury severity score ≥ 15 [27]. In the trauma group, with a mean injury severity score of 27 and a predictive mortality of 35 % [28], the majority (70 %) was subjected to mechanical ventilation and 53 % required vasopressor/norepinephrine infusion, suggesting that this group was a more relevant control group than our healthy controls.

In a recent study, Orde et al. performed speckle tracking echocardiography in adult patients with early severe sepsis/septic shock [29]. The incidence of RV and LV dysfunction was approximately twice as high based on strain analysis when compared to assessment by conventional echocardiography, in their study, suggesting that speckle tracking echocardiography is more sensitive than the conventional technique, a finding that was confirmed in the present study. In the study by Orde et al., however, a control group of non-septic critically ill patients was not included and the cut-off values chosen to define RV and LV dysfunction and severe dysfunction were based on normal subjects at their institution. In a recent paper by De Geer et al., systolic LV function was evaluated by strain echocardiography in 44 patients with septic shock [30]. In contrast to the present study, they found a close correlation (r = 0.70) between longitudinal global LV strain and LV ejection fraction and that in patients with septic shock and preserved LV ejection fraction, only 7 % had a depressed global longitudinal LV strain (< −15 %), compared to 50 % in the present study. This discrepancy could be explained by the fact that they included older patients with a high incidence (48 %) of cardiac co-morbidities compared to the present study.

The LV loading conditions may influence myocardial deformation. Longitudinal LV strain is reduced when afterload is increased, and LV strain is increased when preload is increased [31, 32]. Furthermore, LV strain is sensitive to changes in heart rate. Weidermann et al. [33] showed that a pacing-induced increase in heart rate decreased LV strain. In the present study, LV afterload was lower and heart rate was higher in the septic compared to the trauma group. However, in our multivariate analysis, only heart rate was independently and negatively associated with LV strain, confirming the results of Weidermann et al.

The major limitation of the present study is its retrospective nature and the relative small sample sizes. Another limitation is that three different ultrasound machines were used for the echocardiographic examinations. However, the same software for analysis of strain was used by an experienced operator, to perform all the primary end-point measurements. Furthermore, the observer was blinded to the diagnoses and the intra-observer coefficient of variation was acceptably low.

## Conclusions

Strain echocardiographic LV and RV systolic functions in patients with early severe sepsis and preserved LV ejection fraction were significantly impaired compared to critically ill, non-septic trauma patients. We conclude that strain imaging by speckle-tracking echocardiography may be useful for early detection of septic cardiomyopathy.

## Additional file

Additional file 1: Table S1. ᅟ

## Abbreviations

LV: Left ventricular
RV: Right ventricular
2D: Two-dimensional
TDI: Tissue Doppler imaging
STI: Speckle tracking imaging
ICU: Intensive care unit
PEEP: Positive end-expiratory pressure
FiO_2_: Inspired oxygen fraction
SaO_2_: Arterial oxygen saturation
MAP: Mean arterial pressure
CVP: Central venous pressure
SVR: Systemic vascular resistance
SAPS II: Simplified acute physiology score
Ch: Chamber
TVI-LVOT: Time velocity integral of the LV outflow tract
E-deceleration time: Peak early LV diastolic flow deceleration time
E-max: Maximum flow velocity during early LV diastolic filling
A-max: Maximum flow velocity during late diastolic LV filling
S/D: Ratio between pulmonary vein peak systolic (S) and peak diastolic (D) flow velocities
