# Supplementary material for: Strain echocardiography identifies impaired longitudinal systolic function in patients with septic shock and preserved ejection fraction
Source: Cardiovasc Ultrasound. 2015 Jul 2;13:30. doi: 10.1186/s12947-015-0025-4 (PMC4487964; doi:10.1186/s12947-015-0025-4)
Supplement: Additional file 1: Table S1. — ᅟ [file 12947_2015_25_MOESM1_ESM.docx]

| Additional file 1: TableS1 | | | | | | | |
| --- | --- | --- | --- | --- | --- | --- | --- |
| Pat. | Age (yrs) | Diagnosis | SAPS II | Vasopressor/Inotrope | Length of ICU stay | Organ System Failure (number) | Died |
| 1 | 59 | Urosepsis | 45 | NE | 1 | 1 | No |
| 2 | 70 | Urosepsis | 74 | NE | 5 | 2 | No |
| 3 | 80 | Pneumococcal pneumonia and sepsis | 76 | NE | 6 | 3 | No |
| 4 | 52 | Necrotising Fasciitis | 79 | NE, E | 1 | 4 | Yes |
| 5 | 41 | Beta-hemolytic streptococcus pneumonia and sepsis | 73 | NE, E | 14 | 3 | Yes |
| 6 | 42 | MRSA pneumonia and sepsis | 75 | NE, E | 15 | 3 | No |
| 7 | 46 | Beta-hemolytic streptococcus pneumonia and sepsis | 76 | NE, E | 17 | 3 | No |
| 8 | 36 | Beta-hemolytic streptococcus pneumonia and sepsis | 66 | NE | 9 | 2 | No |
| 9 | 70 | Group A streptococcus pneumonia and sepsis | 48 | NE | 4 | 1 | No |
| 10 | 66 | Stenotrophomonas pneumonia and sepsis | 70 | - | 32 | 3 | No |
| 11 | 60 | Legionella pneumoniophila pneumonia | 47 | - | 14 | 1 | Yes |
| 12 | 32 | Pneumonia and sepsis | 42 | NE | 3 | 1 | No |
| 13 | 46 | Pneumococcal and CNS pneumonia and sepsis | 78 | NE | 54 | 3 | No |
| 14 | 59 | E-coli pneumonia and sepsis | 53 | NE | 4 | 2 | No |
| 15 | 22 | Abdominal sepsis | 72 | - | 5 | 1 | No |
| 16 | 44 | Nekrotised fasciitis | 60 | NE, Mil | 10 | 3 | No |
| 17 | 59 | Staphylococcus aureus pneumonia and sepsis | 68 | NE | 22 | 2 | No |
| 18 | 54 | Aspiration pneumonia | 86 | NE, E | 12 | 2 | No |
| 19 | 55 | ESBL abdominal sepsis | 84 | NE | 4 | 2 | Yes |
| 20 | 32 | Aspiration pneumonia | 51 | NE, Mil | 7 | 2 | No |
| 21 | 28 | Mediastinitis | 34 | NE | 16 | 1 | No |
| 22 | 70 | Abdominal sepsis | 79 | NE | 7 | 2 | No |
| 23 | 50 | Pneumonia | 62 | NE | 3 | 2 | No |
| 24 | 71 | Aspiration pneumonia | 64 | NE | 7 | 2 | No |
| 25 | 34 | Bowel perforation | 46 | NE | 16 | 3 | No |
| 26 | 54 | SBP, E.Coli | 66 | NE | 7 | 2 | No |
| 27 | 70 | Pneumonia | 83 | NE | 4 | 2 | No |
| 28 | 71 | Abdominal sepsis | 95 | NE | 13 | 3 | No |
| 29 | 53 | Pneumonia | 47 | NE | 4 | 2 | No |
| 30 | 69 | Pneumonia | 60 | NE | 7 | 2 | No |
| 31 | 68 | Urosepsis, E.Coli | 74 | NE | 2 | 2 | No |
| 32 | 73 | Necrotising Fasciitis | 77 | NE | 12 | 3 | Yes |
| 33 | 54 | Aspiration pneumonia | 75 | NE | 1 | 2 | No |
| 34 | 64 | Necrotising Fasciitis | 88 | NE, E | 9 | 3 | No |
| 35 | 54 | Bowel perforation | 95 | NE | 4 | 4 | No |
| 36 | 58 | SBP, β-Streptococcus, Candida | 75 | NE | 15 | 3 | No |
| 37 | 63 | Pneumonia, gram-negative sterptococcus | 85 | NE, E, MIL | 16 | 4 | No |
| 38 | 43 | Pneumonococcal pneumonia | 75 | NE, E | 3 | 3 | Yes |
| 39 | 51 | Beta-hemolytic streptococcus group A infection | 80 | NE | 15 | 3 | No |
| 40 | 56 | Pneumonia | 63 | NE | 16 | 3 | No |
| 41 | 54 | Pneumonia | 78 | NE | 13 | 3 | Yes |
| 42 | 56 | Beta-hemolytic streptococcus group A pneumonia | 63 | NE | 15 | 2 | No |
| 43 | 20 | Meningococcal sepsis | 48 | NE | 5 | 2 | No |
| 44 | 57 | Abdominal sepsis | 44 | NE | 40 | 2 | No |
| 45 | 50 | Abdominal sepsis | 80 | NE | 9 | 3 | No |
| 46 | 49 | Peritonitis, Aspergilosis, Zygomycosis | 80 | NE | 3 | 3 | No |
| 47 | 62 | S.Aureus pneumonia | 77 | NE | 2 | 2 | No |
| 48 | 62 | CNS sepsis | 65 | NE | 10 | 2 | No |

ICU; intensive care unit, MRSA = Methicillin-resistant staphylococcus aureus, E-coli = Escherichia-coli, CNS = Coagulase-negative staphylococcus, ESBL = Extended spectrum beta-lactamase, SBP = Spontaneous bacterial peritonitis, NE; norepinephrine, E; epinephrine, Mil; milrinone
